# Genetically predicted CXCL16 expression is associated with Parkinson’s disease risk and peripheral immune cell dysregulation: a two-sample mendelian randomization study

**DOI:** 10.1186/s13041-026-01324-z

**Published:** 2026-06-30

**Authors:** Zhou Li, Rong Yang, Hao Feng, Mou Sun, Hongjun Liu

**Affiliations:** 1 Beijing Anzhen Nanchong Hospital of Capital Medical University & Nanchong Central Hospital, Nanchong, 637000 Sichuan China; 2 Department of Neurosurgery, Nanchong Central Hospital, The Second Clinical Medical College, North Sichuan Medical College, Nanchong, 637000 Sichuan China; 3 Department of Neurosurgery, Affiliated Hospital of North Sichuan Medical College, Nanchong, 637000 Sichuan China

**Keywords:** Parkinson’s disease, PANoptosis, Mendelian randomization, Immune cells, CXCL16, Neuroinflammation, eQTL

## Abstract

**Background:**

Parkinson’s disease (PD) is a progressive neurodegenerative disorder with limited disease-modifying therapies. PANoptosis, an integrated form of programmed cell death involving apoptosis, pyroptosis, and necroptosis, has been implicated in neuroinflammation-related neurodegeneration. However, the roles of PANoptosis-related genes in PD remain unclear.

**Methods:**

We performed two-sample Mendelian randomization (MR) using cis-eQTL instruments from the eQTLGen Consortium for 30 PANoptosis-related genes, with PD GWAS data from Nalls et al. 2019 as the outcome. Instrumental variables were selected using a hierarchical strategy, with genome-wide significant cis-eQTLs as primary instruments and a relaxed threshold applied only for genes with fewer than three independent SNPs. Sensitivity analyses included MR-Egger, weighted median, MR-PRESSO, MR-RAPS, and leave-one-out analyses. SMR/HEIDI testing and two-step MR mediation using 731 peripheral immune traits were also performed.

**Results:**

Genetically predicted higher CXCL16 expression was associated with increased PD risk (OR = 1.115, 95% CI 1.060–1.173, *p* = 2.4 × 10^−5^), while higher FADD expression was associated with reduced PD risk (OR = 0.861, 95% CI 0.790–0.939, *p* = 7.1 × 10^−4^). CASP1 and IFI27 were nominally significant and considered exploratory. Sensitivity analyses were directionally consistent, although MR-Egger estimates were imprecise. SMR/HEIDI supported CXCL16. Exploratory mediation analysis identified 63/66 candidate immune mediators after FDR correction.

**Conclusion:**

These findings provide MR-based genetic evidence linking CXCL16 expression to PD risk, with exploratory mediation through peripheral immune phenotypes. The CXCL16–immune cell–PD axis warrants further experimental validation.

**Graphical abstract:**

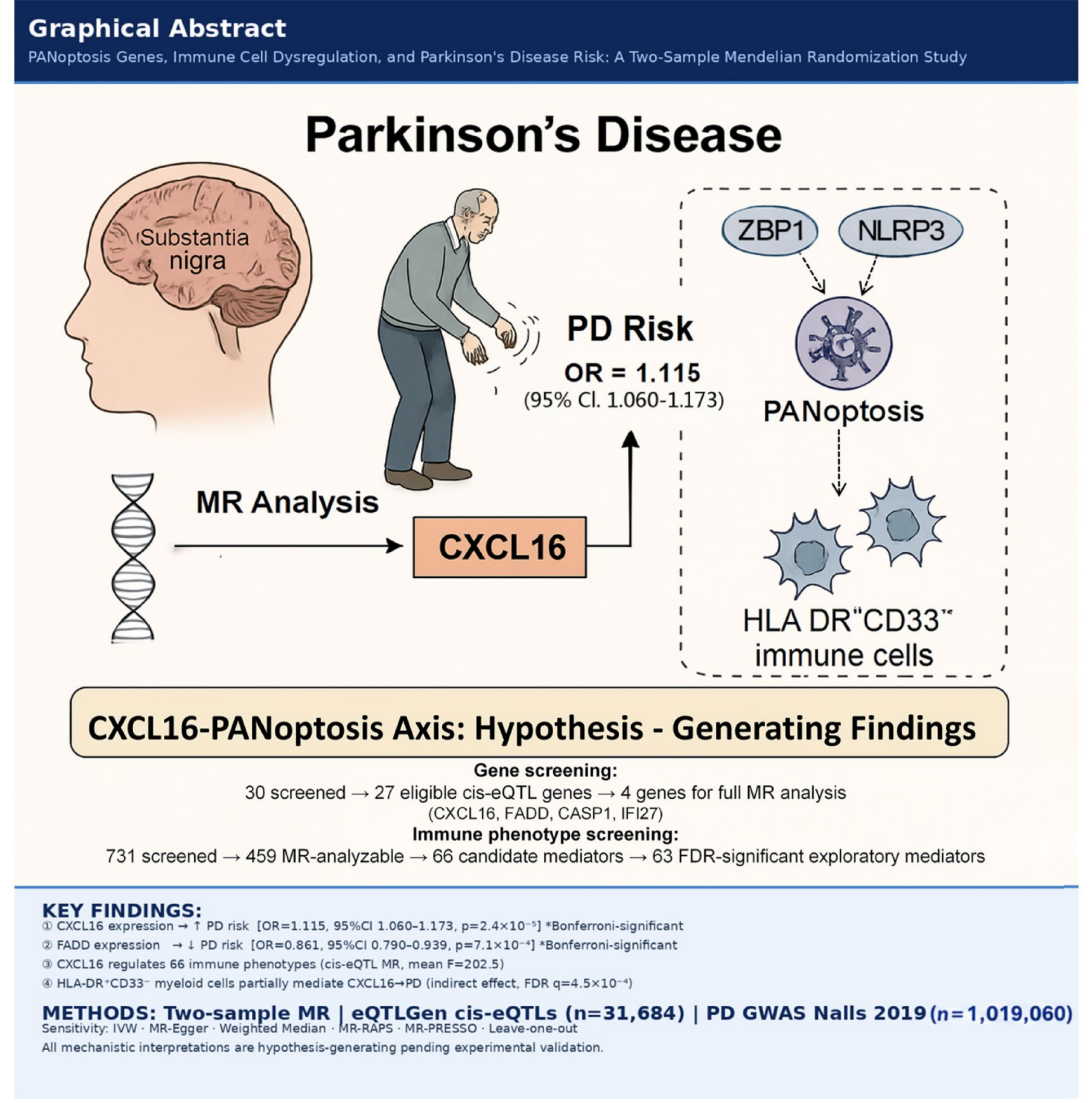

**Supplementary Information:**

The online version contains supplementary material available at 10.1186/s13041-026-01324-z.

## Introduction

Parkinson’s disease (PD) is a progressive neurodegenerative condition characterized primarily by motor impairment. According to the 2021 Global Burden of Disease database, worldwide PD prevalence escalated from approximately 450,000 cases in 1992 to 1.34 million by 2021, with Age-Standardized Incidence Rate rising from 11.54 to 15.63 per 100,000 individuals [1]. Incidence among individuals aged 55 and older surged from 2.83 million in 1990–10.76 million in 2021, and annual new diagnoses increased 219% over the same period [2]. PD is pathologically defined by degeneration of dopaminergic neurons in the substantia nigra, manifesting as tremors, rigidity, and bradykinesia [3]. Current therapies provide symptomatic relief but do not halt disease progression [4, 5], underscoring the need for novel targets.

PANoptosis is a recently described form of programmed cell death that simultaneously engages apoptosis, pyroptosis, and necroptosis components, coordinated by a multimolecular PANoptosome complex [6–10]. Key regulators include ZBP1, NLRP3, FADD, CASP1, CASP8, RIPK3, and GSDMD. Emerging evidence from PD-related experimental models and neuroinflammation studies implicates dysregulated inflammatory cell-death pathways in neurodegenerative processes [11, 12], though causal relationships have not been established.

Mendelian randomization (MR) leverages germline genetic variants as instrumental variables to estimate causal effects while reducing confounding and reverse causality [13]. Expression quantitative trait loci (eQTLs) enable inference about gene expression effects. Here we performed two-sample MR to evaluate genetic association between 30 PANoptosis gene expressions and PD risk, followed by SMR/HEIDI validation and two-step MR mediation through immune cell phenotypes. All mechanistic interpretations are presented as hypothesis-generating (Supplementary Fig. S1).

## Methods

### Study design

This study employed a two-sample MR framework. The analysis proceeded in three stages: (i) primary MR of 30 PANoptosis gene expressions against PD risk; (ii) SMR/HEIDI analysis for eQTL–GWAS colocalization; and (iii) two-step MR mediation through 731 immune phenotypes. Reporting follows STROBE-MR guidelines (Supplementary Checklist S1).

### Data sources

Exposure (PANoptosis eQTLs): cis-eQTL data from eQTLGen Consortium (n = 31,684 whole-blood; https://eqtlgen.org) [14]. Cis-eQTLs were defined as variants within ± 1 Mb of gene TSS. Thirty PANoptosis genes were selected from published literature and pathway databases [7]; the complete list is in Supplementary Table S1.

Outcome (PD GWAS): Primary outcome was from Nalls et al. 2019 [15] (37,688 PD cases, 981,372 controls, European ancestry; IEU OpenGWAS). Secondary validation used FinnGen R12 (5861 PD cases, 494,487 controls; finngen_R12_G6_PARKINSON).

Immune phenotypes: Genetic associations for 731 immune traits from Orrù et al. 2020 [16] (IEU OpenGWAS; n ≈ 22,000), covering B cells, T cells, monocytes, dendritic cells, and granulocytes (Supplementary Table S2). All datasets are publicly available with prior institutional ethical approval; no additional ethics review was required.

### Instrumental variable selection

Genome-wide significant cis-eQTLs (*p* < 5 × 10^−8^) were selected as the primary instrumental variables. For genes with fewer than three independent SNPs at this threshold, a relaxed cis-eQTL threshold of *p* < 1 × 10^−5^ was applied to improve instrument availability. Independent SNPs were retained after LD clumping (r^2^ < 0.001, window = 10,000 kb) using the 1000 Genomes EUR reference panel. All retained SNPs were cis-eQTLs within ± 1 Mb of the TSS. Instrument strength was assessed by F-statistic (F = [beta/SE]^2^) and R^2^; all instruments far exceeded the weak-instrument threshold of F > 10 (Supplementary Table S3). Palindromic SNPs with MAF < 0.42 were retained with EAF alignment (action = 2), whereas ambiguous palindromic SNPs with MAF ≥ 0.42 were excluded. Steiger filtering was performed to confirm the exposure-to-outcome direction.

### Primary MR analysis

MR was conducted using TwoSampleMR (v0.6.0) [17]. The primary estimator was inverse variance weighted (IVW) under random effects. Supplementary methods: MR-Egger, weighted median, weighted mode, and simple mode [18–20]. Results are expressed as OR per one SD increase in genetically predicted gene expression with 95% CI. For genes with a single SNP, the Wald ratio was used. Bonferroni correction was applied across the four analyzable primary MR tests because only four genes had complete harmonized PD GWAS data for full MR analysis. The primary significance threshold was therefore set at *p* < 0.0125. For transparency, a more conservative correction across all 30 initially screened PANoptosis genes was also reported, with a threshold of *p* < 0.00167.

### Sensitivity analyses

Heterogeneity: Cochran’s Q test. Directional pleiotropy: MR-Egger intercept. Outlier pleiotropy: MR-PRESSO global test [21]; outlier-corrected estimates reported when applicable. Leave-one-out analysis was performed by sequentially excluding each SNP. MR-RAPS (Robust Adjusted Profile Score, Huber loss function) [22] was applied as an additional weak-instrument-robust sensitivity check.

### SMR/HEIDI analysis

SMR (v1.3.1; https://cnsgenomics.com/software/smr/) tested whether genetic variants associated with PANoptosis gene expression also affect PD risk through a shared causal variant [23]. The HEIDI test distinguishes pleiotropy from linkage (HEIDI *p* > 0.05 supports a single shared causal variant). Full locus-level colocalization using the coloc framework was not feasible due to the absence of full locus-level summary statistics from eQTLGen; HEIDI results serve as an approximate colocalization proxy.

### Mediation analysis

Two-step MR mediation analysis was performed following Burgess et al. [24]. In Step 1, we estimated the association of genetically predicted CXCL16 expression with each immune trait (β₁, SE₁). In Step 2, we estimated the association of each candidate immune trait with PD risk (β₂, SE₂). The indirect effect was calculated as β₁ × β₂, with the delta-method standard error estimated as SE_indirect = √(β₁^2^ × SE₂^2^ + β₂^2^ × SE₁^2^). A Z-statistic and two-sided p-value were calculated for each mediator. The proportion mediated was estimated as (β₁ × β₂) / β_total, where β_total represents the total MR estimate for CXCL16 expression on PD risk.

For immune phenotype filtering, 731 peripheral immune traits were initially screened. Among them, 459 traits had sufficient SNP overlap and harmonized data for MR analysis. Immune traits showing nominally significant associations with genetically predicted CXCL16 expression in Step 1 MR (*p* < 0.05) were selected as candidate mediators. FDR correction was then applied across these candidate mediators. All mediation findings were interpreted as exploratory.

## Results

### Instrumental variable characteristics

Of 30 PANoptosis genes, 27 had at least one cis-eQTL available for instrument construction (GSDME: none; ZBP1, RIPK3, HSP90AB1: ≤ 2 SNPs at the primary threshold). Instruments per gene ranged from 1 (HSP90AB1) to 41 (MLKL). Instrumental variables were selected using a hierarchical threshold strategy: genome-wide significant cis-eQTLs at *p* < 5 × 10^−8^ were used as the primary instruments, and for genes with fewer than three independent SNPs at this threshold, a relaxed cis-eQTL threshold of *p* < 1 × 10^−5^ was applied to improve instrument availability. After LD clumping and harmonization with the PD GWAS dataset, four genes had complete harmonised PD GWAS data for full MR analysis: CXCL16 (nSNP = 37), CASP1 (nSNP = 13), FADD (nSNP = 17), and IFI27 (nSNP = 13).

All instruments were strong. Mean F-statistics: CXCL16 = 202.5 (R^2^ = 0.0071), CASP1 = 158.7 (R^2^ = 0.0052), FADD = 154.4 (R^2^ = 0.0049), IFI27 = 107.1 (R^2^ = 0.0035). Across all 26 multi-SNP genes, mean F ranged from 64.6 to 359.3, all substantially exceeding F = 10 (Supplementary Table S3). Steiger filtering confirmed causal direction for all retained SNPs.

### Primary MR analysis: PANoptosis genes and PD risk

**Fig. 1:**
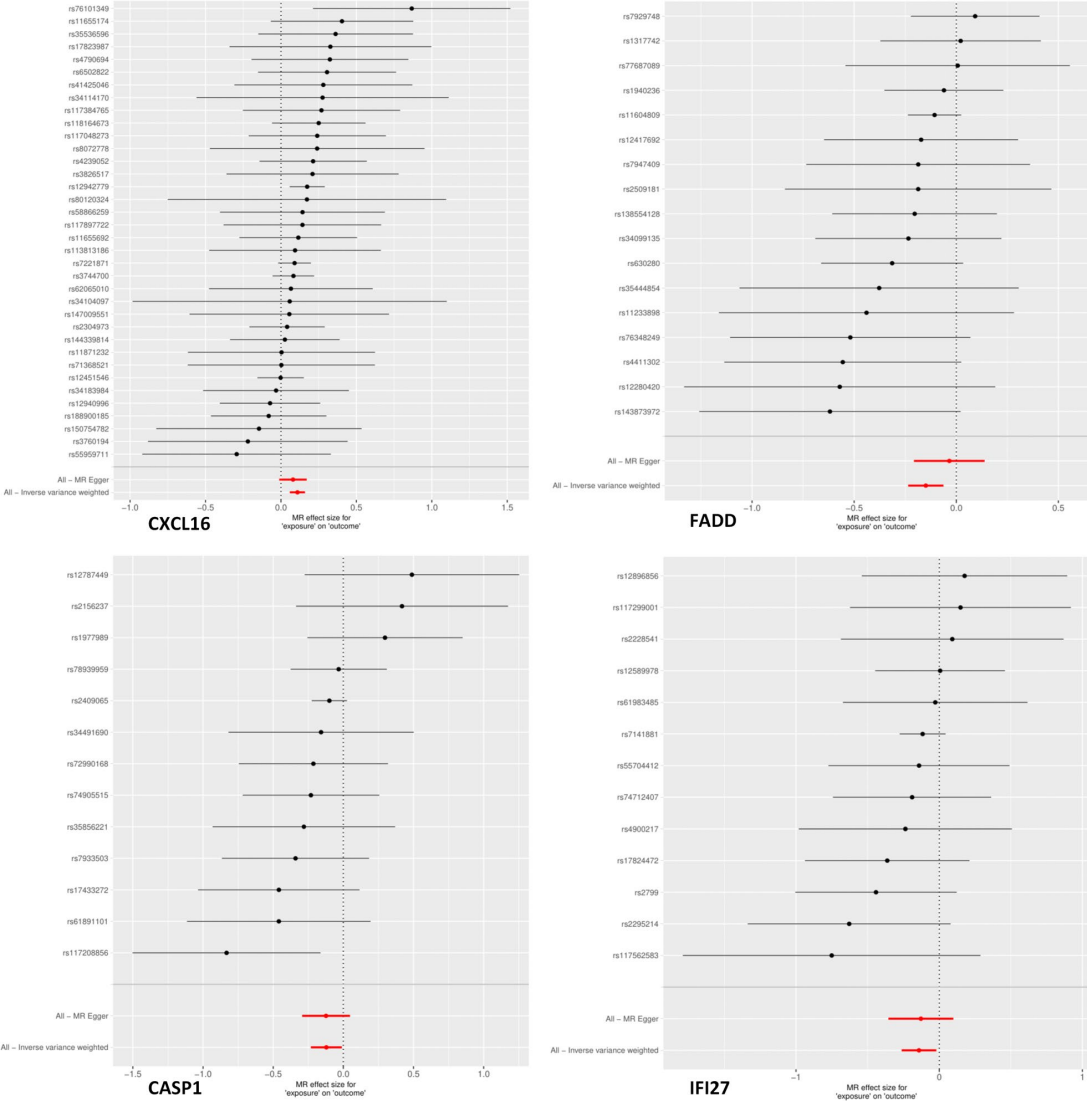
Forest plot of inverse variance weighted (IVW) Mendelian randomization analysis for four PANoptosis gene expressions and Parkinson’s disease (PD) risk. Each point represents the IVW estimate (OR, 95% CI) per 1-SD increase in genetically predicted gene expression. Dashed vertical line = null (OR = 1.0). Instrumental variables: genome-wide significant cis-eQTLs (*p* < 5 × 10^−8^) from eQTLGen Consortium (n = 31,684). Bonferroni threshold for 4 primary tests: *p* < 0.0125. *Bonferroni-significant: CXCL16 (OR = 1.115, *p* = 2.4 × 10^−5^) and FADD (OR = 0.861, *p* = 7.1 × 10^−4^). †Nominally significant (exploratory): CASP1 (*p* = 0.032), IFI27 (*p* = 0.022). Note: Summary-level IVW estimates are shown; detailed MR sensitivity analyses for CXCL16 and FADD are provided in Figs. 2–3, and those for CASP1 and IFI27 are provided in Supplementary Figures S2, S3

IVW analysis identified four nominally significant PANoptosis gene–PD associations (Fig. 1, Supplementary Table S4). Genetically predicted higher CXCL16 expression was associated with increased PD risk (OR = 1.115, 95% CI 1.060–1.173, *p* = 2.4 × 10^−5^), whereas higher genetically predicted expression of FADD (OR = 0.861, 95% CI 0.790–0.939, *p* = 7.1 × 10^−4^), CASP1 (OR = 0.886, 95% CI 0.794–0.989, *p* = 0.032), and IFI27 (OR = 0.868, 95% CI 0.769–0.980, *p* = 0.022) were associated with reduced PD risk. Following Bonferroni correction for the four primary MR tests (threshold *p* < 0.0125), CXCL16 (*p*_Bonf = 9.7 × 10^−5^) and FADD (*p*_Bonf = 2.9 × 10^−3^) remained statistically significant, while CASP1 and IFI27 did not survive correction and are designated as exploratory findings. To ensure transparency regarding our initial discovery framework, we also performed a conservative Bonferroni correction across all 30 screened PANoptosis genes (threshold *p* < 0.00167); under this stringent threshold, CXCL16 (*p*_Bonf = 7.2 × 10^−4^) remained significant, whereas FADD was borderline (*p*_Bonf = 0.021).

### Sensitivity analyses

Results were directionally consistent across IVW, weighted median, MR-RAPS, and MR-PRESSO for CXCL16 and FADD, although MR-Egger estimates were imprecise and non-significant (Table 2, Supplementary Table S5). For CXCL16 (nSNP = 36, Mean F = 202.5): MR-Egger OR = 1.083 (95% CI 0.988–1.187, *p* = 0.097); weighted median OR = 1.094 (95% CI 1.018–1.175, *p* = 0.014); MR-PRESSO raw OR = 1.115 (global test *p* = 0.963, no outliers); MR-RAPS OR = 1.114 (95% CI 1.057–1.174, *p* = 5.2 × 10^−5^). MR-Egger intercept: 0.0067 (*p* = 0.456, no pleiotropy). Cochran Q = 21.7 (df = 35, *p* = 0.961, no heterogeneity).

**Fig. 2:**
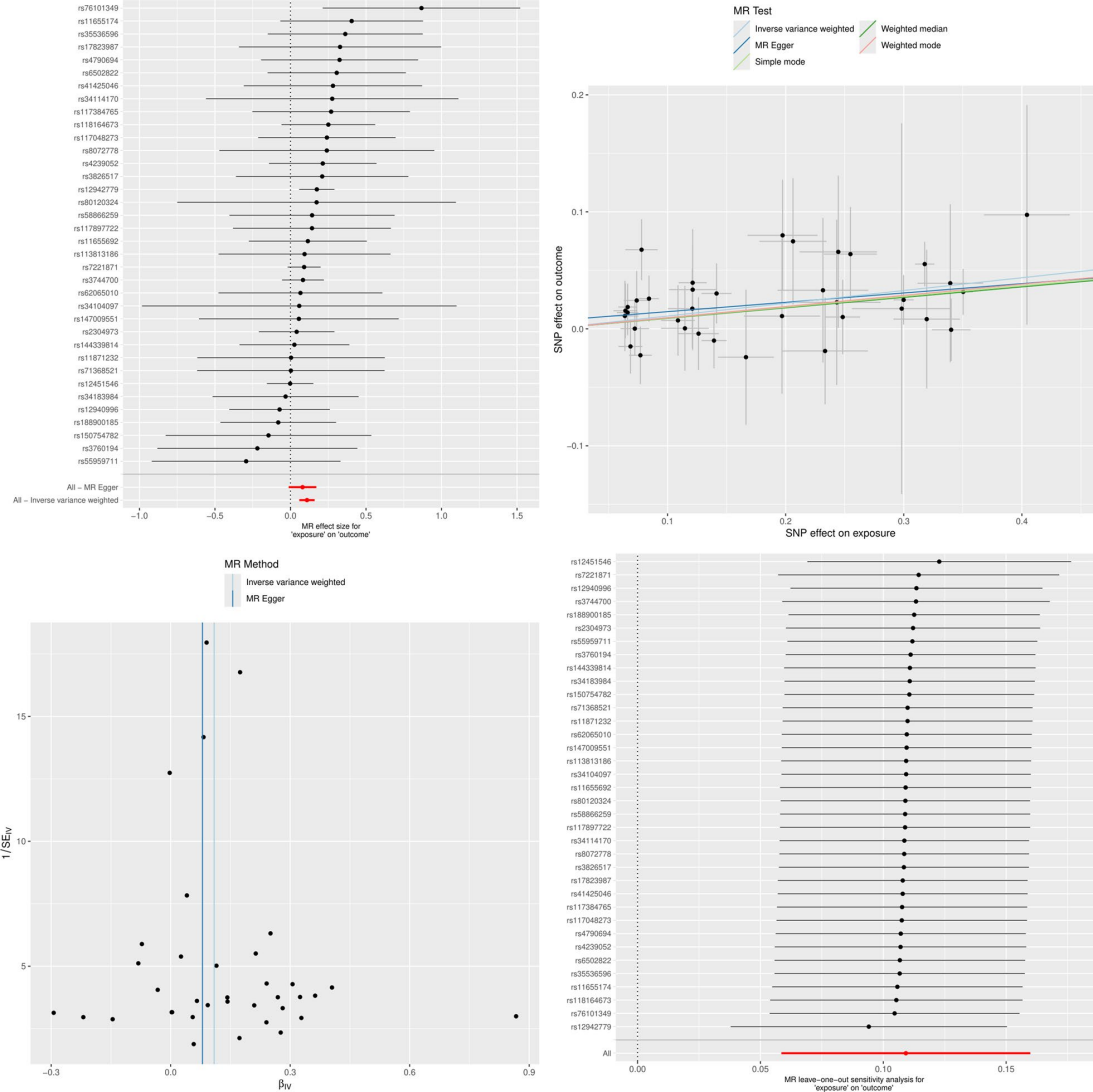
MR sensitivity analysis for CXCL16 → PD (nSNP = 36, mean F = 202.5, mean R^2^ = 0.0071). Top-left: Forest plot. Top-right: Scatter plot. Bottom-left: Funnel plot. Bottom-right: Leave-one-out analysis. IVW: OR = 1.115 [1.060–1.173], *p* = 2.4 × 10^−5^. MR-Egger intercept *p* = 0.456 (no pleiotropy). MR-PRESSO Global *p* = 0.963 (no outliers). Cochran Q *p* = 0.961 (no heterogeneity)

**Fig. 3:**
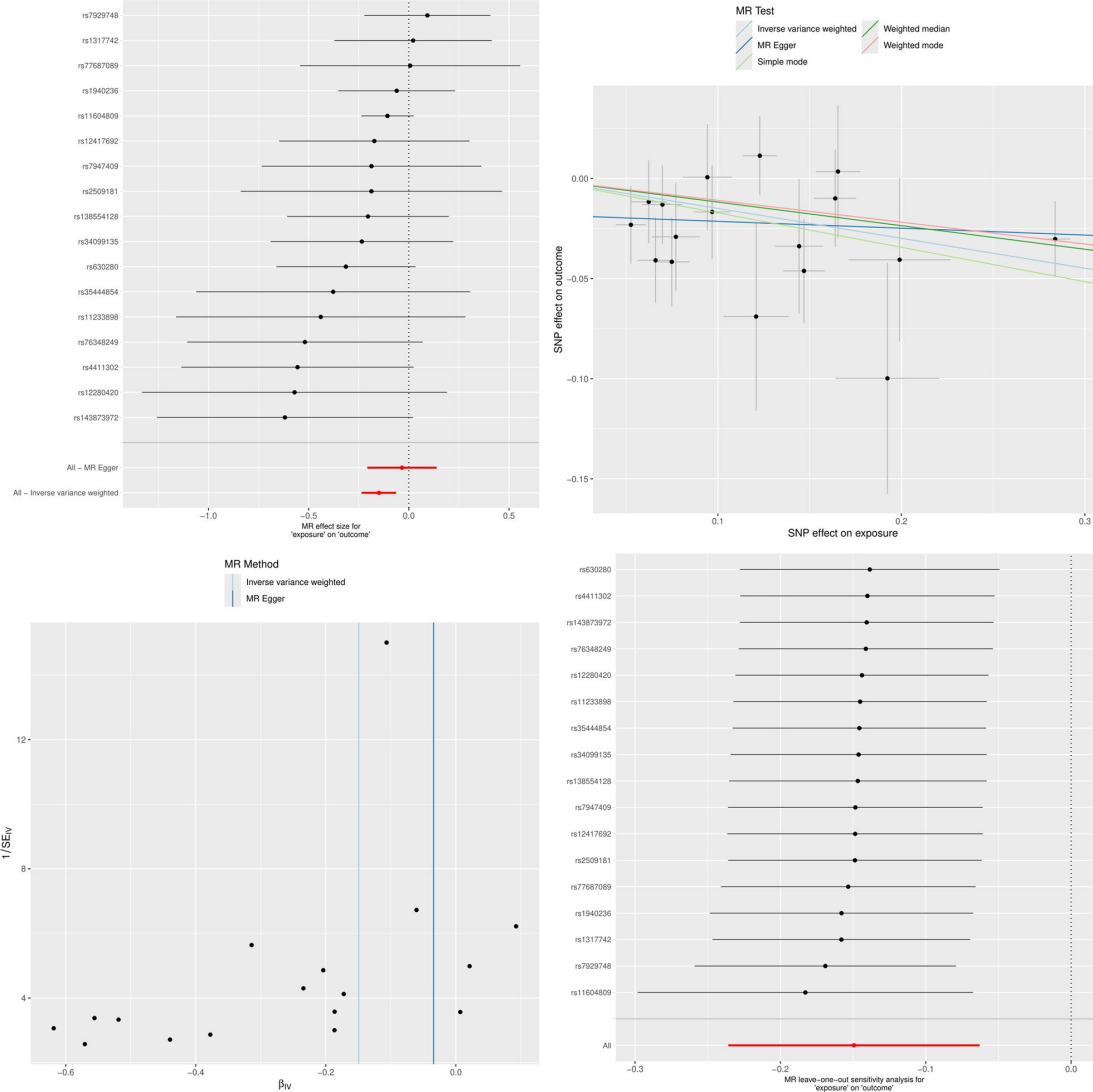
MR sensitivity analysis for FADD → PD (nSNP = 17, mean F = 154.4, mean R^2^ = 0.0049). Top-left: Forest plot. Top-right: Scatter plot. Bottom-left: Funnel plot. Bottom-right: Leave-one-out analysis. IVW: OR = 0.861 [0.790–0.939], *p* = 7.1 × 10^−4^. MR-Egger intercept *p* = 0.154 (no pleiotropy). MR-PRESSO Global *p* = 0.699 (no outliers). Cochran Q *p* = 0.683 (no heterogeneity)

For FADD (nSNP = 17, Mean F = 154.4): MR-Egger OR = 0.966 (95% CI 0.813–1.149, *p* = 0.704); weighted median OR = 0.889 (95% CI 0.794–0.996, *p* = 0.042); MR-PRESSO OR = 0.861 (global test *p* = 0.699, no outliers); MR-RAPS OR = 0.859 (95% CI 0.786–0.940, *p* = 9.3 × 10^−4^). No heterogeneity (Q = 12.9, df = 16, *p* = 0.683) or pleiotropy (intercept *p* = 0.154). Leave-one-out analyses showed no single SNP drove the observed associations in the four analyzed genes (Figs. 2–3, Supplementary Figs. S2, S3).

### SMR/HEIDI analysis

CXCL16 was the only PANoptosis gene reaching SMR significance (b-SMR = 0.124, SE = 0.043, *p*-SMR = 0.004; Table 1). HEIDI *p* = 0.363 (> 0.05) indicates consistency with a shared causal variant, rather than LD confounding. FADD (*p*-SMR = 0.110), CASP1 (*p*-SMR = 0.122), and IFI27 (*p*-SMR = 0.157) did not reach SMR significance.

**Table 1:** SMR analysis of PANoptosis gene associations with Parkinson’s disease

Gene	b-SMR	SE	*p*-SMR	*p*-HEIDI	nSNP-HEIDI
CXCL16	0.124	0.043	0.004*	0.363	20
FADD	−0.109	0.068	0.110	0.581	20
CASP1	−0.101	0.065	0.122	0.502	17
IFI27	−0.117	0.083	0.157	0.966	20

*p < 0.05; HEIDI *p* > 0.05 indicates consistency with a single shared causal variant; SMR = summary-data-based Mendelian randomization; HEIDI = Heterogeneity in Dependent Instruments

### Immune cell phenotypes and PD risk

**Fig. 4:**
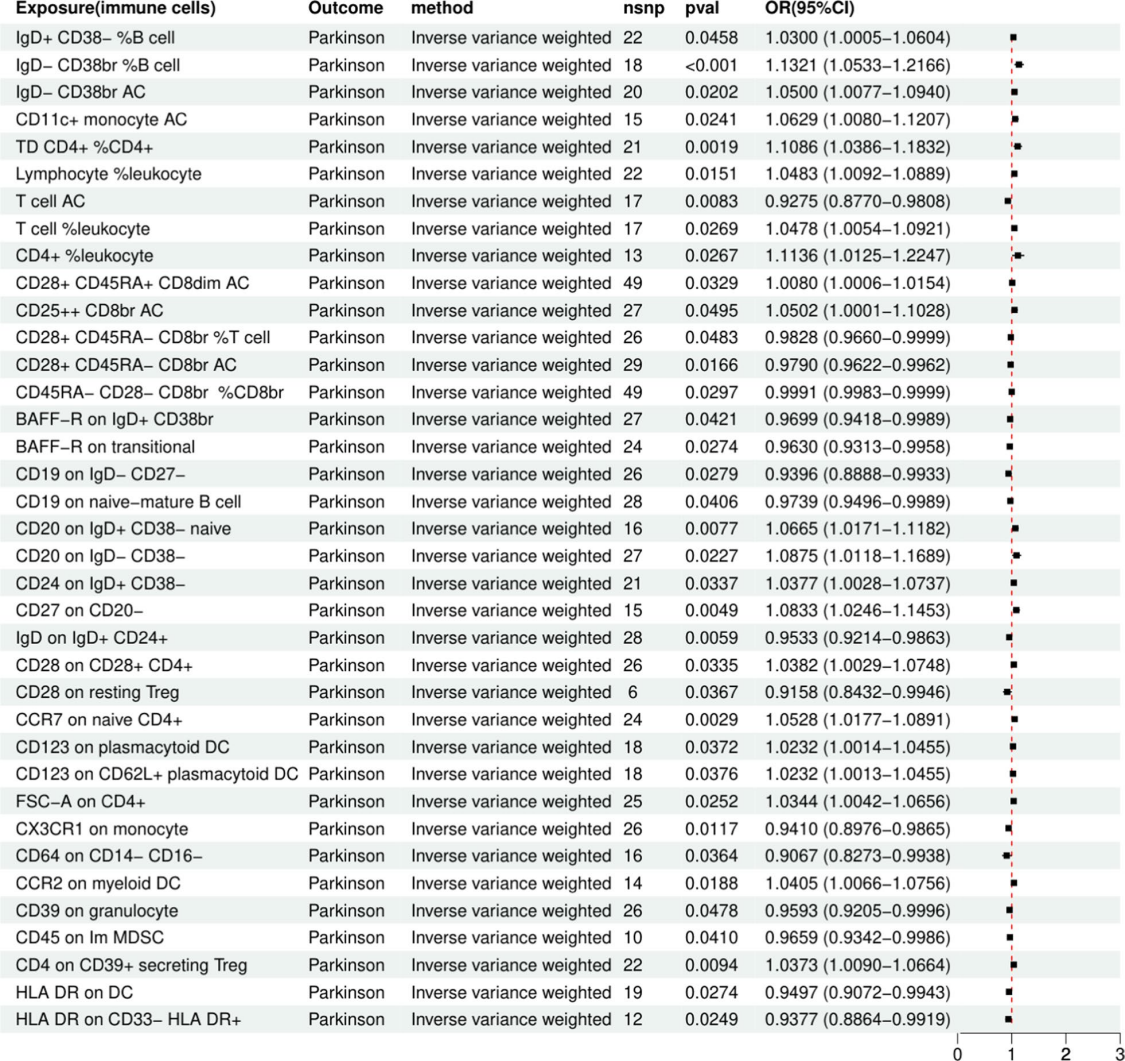
Mendelian randomization analysis (IVW method) of significant immune cell phenotype associations with Parkinson’s disease risk. OR per 1-SD change in immune trait level (95% CI). Selected nominally significant associations (*p* < 0.05) from 459 immune traits (Orrù et al. 2020) tested against PD GWAS (Nalls et al. 2019). Risk-associated: IgD⁻CD38br⁺ B cells, CCR7⁺ naive CD4⁺ T cells, CD11c⁺ monocytes. Protective: CX3CR1⁺ monocytes, HLA-DR⁺CD33⁻ cells. Full results: Supplementary Table S5. Data from 459 immune traits (Orrù et al. 2020, n ≈ 22,000). All immune traits reflect peripheral blood. Note: Results reflect nominally significant associations (*p* < 0.05); full FDR-corrected results are provided in Supplementary Table S5

Of 731 immune traits tested, 459 had sufficient SNP overlap for MR analysis. Nominally significant associations (*p* < 0.05) included risk-associated phenotypes: IgD⁻CD38br⁺ B cells (OR = 1.132, 95% CI 1.053–1.217), CCR7⁺ naive CD4⁺ T cells (OR = 1.053, 95% CI 1.018–1.089), and CD11c⁺ monocytes (OR = 1.063, 95% CI 1.008–1.121); and protective phenotypes: CX3CR1⁺ monocytes (OR = 0.941, 95% CI 0.898–0.987) and HLA-DR⁺CD33⁻ cells (OR = 0.938, 95% CI 0.886–0.992). All results are in Supplementary Table S5. These immune traits reflect peripheral blood and do not directly measure CNS immune activity (Fig. 4).

### CXCL16 expression and immune cell phenotypes

**Fig. 5:**
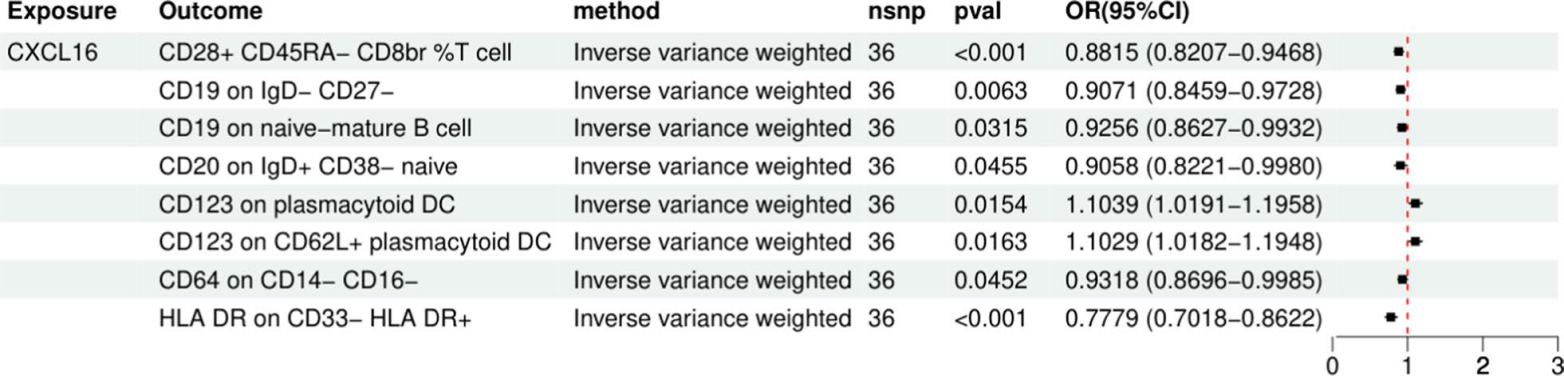
Mendelian randomization analysis (IVW method) for genetically predicted CXCL16 expression on immune cell phenotypes (Step 1 of mediation). OR per 1-SD increase in CXCL16 (95% CI). IVs: 37 GWS cis-eQTLs, mean F = 202.5. Nominally significant (*p* < 0.05) shown; 66 traits used as candidate mediators. Full results: Supplementary Table S6. Data from 731 screened traits; 459 had sufficient SNP overlap; 66 reached *p* < 0.05 and were used as candidate mediators. Note: Results reflect nominally significant associations (*p* < 0.05). Full nominal and FDR-corrected results are provided in Supplementary Table S6

Genetically predicted CXCL16 expression was associated with 66 immune traits at nominal significance (*p* < 0.05; Supplementary Table S6, Fig. 5). Higher CXCL16 was associated with lower HLA-DR⁺CD33⁻ cells (OR = 0.778, 95% CI 0.702–0.862) and CD28⁺CD45RA⁻CD8br⁺ T cells (OR = 0.882, 95% CI 0.821–0.947), and with increased CD123 on plasmacytoid dendritic cells (OR = 1.103–1.104). These are genetic associations; specific molecular mechanisms are not established by this analysis.

### Mediation analysis: CXCL16 → immune traits → PD

Among 731 peripheral immune traits initially screened, 459 were MR-analyzable after SNP overlap and harmonization. Two-step MR mediation was performed for all 66 CXCL16-associated candidate immune traits selected at nominal significance in Step 1 MR (Supplementary Table S7). Indirect effects were estimated using delta-method SEs. After FDR correction across these 66 candidate mediators, 63 indirect effects met *q* < 0.05. The strongest exploratory indirect effect was observed for HLA-DR⁺CD33⁻ cells: indirect effect = 0.0082 (SE = 0.0018, 95% CI 0.0046–0.0117, *p* = 6.7 × 10^−6^, FDR q = 4.5 × 10^4^), representing approximately 7.5% of the total CXCL16–PD MR estimate. The small absolute magnitude of the indirect effect, equivalent to an approximate OR of 1.008 for the top mediator, suggests that these mediation findings should be interpreted as exploratory and hypothesis-generating. The indirect effects, mediator heatmap, and top mediation pathway are shown in Supplementary Figures S4–S6.

**Table 2:** MR sensitivity analysis results for primary PANoptosis gene–PD associations

Gene	Method	nSNP	OR	95% CI	*p*-value
CXCL16	IVW	36	1.115	1.060–1.173	2.4 × 10^−5^*
	MR-Egger	36	1.083	0.988–1.187	0.097
	Weighted Median	36	1.094	1.018–1.175	0.014
	MR-RAPS	36	1.114	1.057–1.174	5.2 × 10^−5^
	MR-PRESSO (raw)	36	1.115	—	Global p = 0.963
FADD	IVW	17	0.861	0.790–0.939	7.1 × 10^−4^*
	MR-Egger	17	0.966	0.813–1.149	0.704
	Weighted Median	17	0.889	0.794–0.996	0.042
	MR-RAPS	17	0.859	0.786–0.940	9.3 × 10^−4^
	MR-PRESSO (raw)	17	0.861	−	Global *p* = 0.699
CASP1^a^	IVW	13	0.886	0.794–0.989	0.032
IFI27^a^	IVW	13	0.868	0.769–0.980	0.022

*Bonferroni-significant (*p* < 0.0125 for 4-gene correction). ^a^Nominally significant only; exploratory. IVW = inverse variance weighted; MR-RAPS = robust adjusted profile score; MR-PRESSO = Mendelian Randomization Pleiotropy RESidual Sum and Outlier

## Discussion

This study provides MR-based genetic evidence that genetically predicted CXCL16 and FADD expression are associated with PD risk. These associations were directionally consistent across IVW, weighted median, MR-RAPS, and MR-PRESSO, and the CXCL16 finding was further supported by SMR/HEIDI. CASP1 and IFI27 showed only nominal significance and should be regarded as exploratory findings requiring independent replication.

CXCL16 is a transmembrane chemokine involved in immune cell recruitment through CXCR6. Chemokine-mediated immune recruitment and neuroinflammatory pathways have been implicated in neurodegenerative diseases [25]. Our findings are consistent with these observations and suggest that genetically elevated CXCL16 may be associated with PD risk partly through peripheral immune-related pathways, although the underlying biological mechanisms remain to be experimentally validated. CXCL16 has also been implicated in inflammatory and vascular injury-related contexts [26–28]. In the present study, the observed effect size was modest but warrants further investigation in PD-relevant tissues and experimental models.

FADD and CASP1 play critical roles in apoptosis and pyroptosis regulation, respectively. FADD inhibits excessive necroptosis through death receptor signaling [29, 30], while CASP1 activation cleaves gasdermin D, inducing pyroptosis that may clear dysfunctional neurons [31, 32]. The protective directions observed for these genes may be compatible with a role of PANoptosis-related regulation, but these exploratory associations require independent replication and experimental validation. IFI27 has been implicated in antiviral and immune-regulatory responses [33], although its specific role in PD and PANoptosis-related immune regulation remains unclear. Immune dysregulation involving innate and adaptive immune cells has been repeatedly reported in PD [34–42].

Our exploratory mediation analysis suggested partial mediation through HLA-DR⁺CD33⁻ myeloid cells, a population with antigen-presenting and immunoregulatory functions. However, the immune phenotype data are from peripheral blood (Orrù et al. 2020) and do not directly capture neuroinflammation or CNS immune activity. References to microglial or central immune dysregulation based on these data are speculative and require independent validation using CSF or brain-tissue-derived immune phenotype data [43].

The proposed involvement of ZBP1–NLRP3–PANoptosis pathways [44, 45] represents a testable hypothesis consistent with our genetic findings but is not directly established by this MR study. Experimental validation, including cell-based assays and PD animal models, is required. All mechanistic statements in this manuscript are hypothesis-generating.

Limitations: (1) Blood eQTLs were used as proxies; brain-specific expression patterns may differ, and sufficiently powered brain eQTL datasets are unavailable for all 30 genes. (2) Formal locus-level colocalization (coloc) was not feasible due to absence of full locus-level eQTLGen summary statistics; HEIDI was used as an approximation. (3) Immune phenotypes reflect peripheral blood only. (4) Analyses were conducted in European-ancestry populations, limiting generalizability. (5) Wet-laboratory validation of identified associations is required.

## Conclusion

This two-sample MR study provides genetic evidence that genetically predicted CXCL16 expression is associated with increased PD risk and that genetically predicted FADD expression is associated with reduced PD risk. The CXCL16 association was directionally consistent across several sensitivity analyses and supported by SMR/HEIDI. Exploratory mediation analysis suggested partial mediation through peripheral immune cell phenotypes. These findings generate testable hypotheses about PANoptosis-related immune mechanisms in PD and highlight the CXCL16–peripheral immune cell axis as a candidate direction for future experimental validation.

## Supplementary Information

Supplementary Material 1.

Supplementary Material 2.

Supplementary Material 3.

Supplementary Material 4.

Supplementary Material 5.

Supplementary Material 6.

Supplementary Material 7.

## Data Availability

All data are publicly available. eQTLGen: https://eqtlgen.org. PD GWAS (Nalls et al. 2019): IEU OpenGWAS (id: ieu-b-7). Immune cell GWAS (Orrù et al. 2020): IEU OpenGWAS. FinnGen R12: https://www.finngen.fi. Analysis code available on request.
