# Supplementary figures and images for: Genetically predicted CXCL16 expression is associated with Parkinson’s disease risk and peripheral immune cell dysregulation: a two-sample mendelian randomization study
Source: Mol Brain. 2026 Jun 30;19:52. doi: 10.1186/s13041-026-01324-z (PMC13321530; doi:10.1186/s13041-026-01324-z)

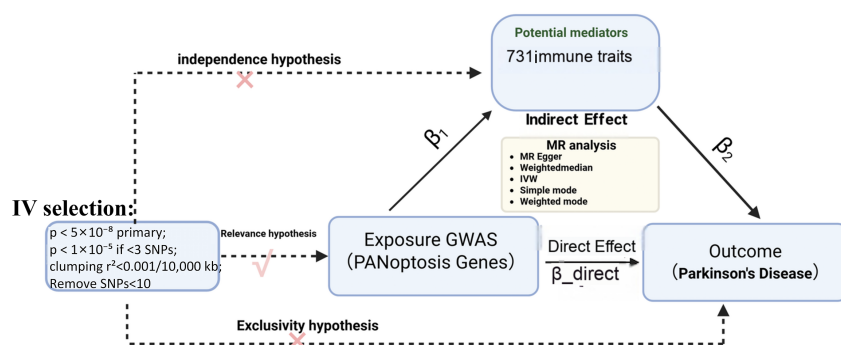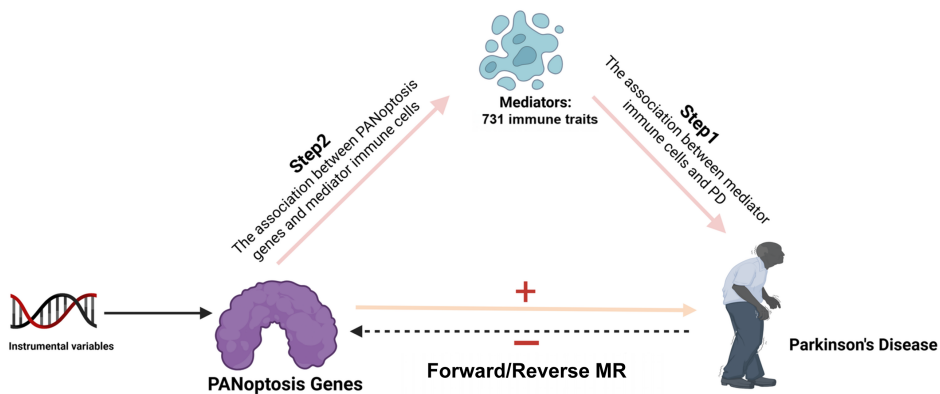

Supplement: Supplementary file 1 — Supplementary Material 1. [file 13041_2026_1324_MOESM1_ESM.pdf]

### Mediation Analysis Heatmap

\* indicates  $p < 0.05$

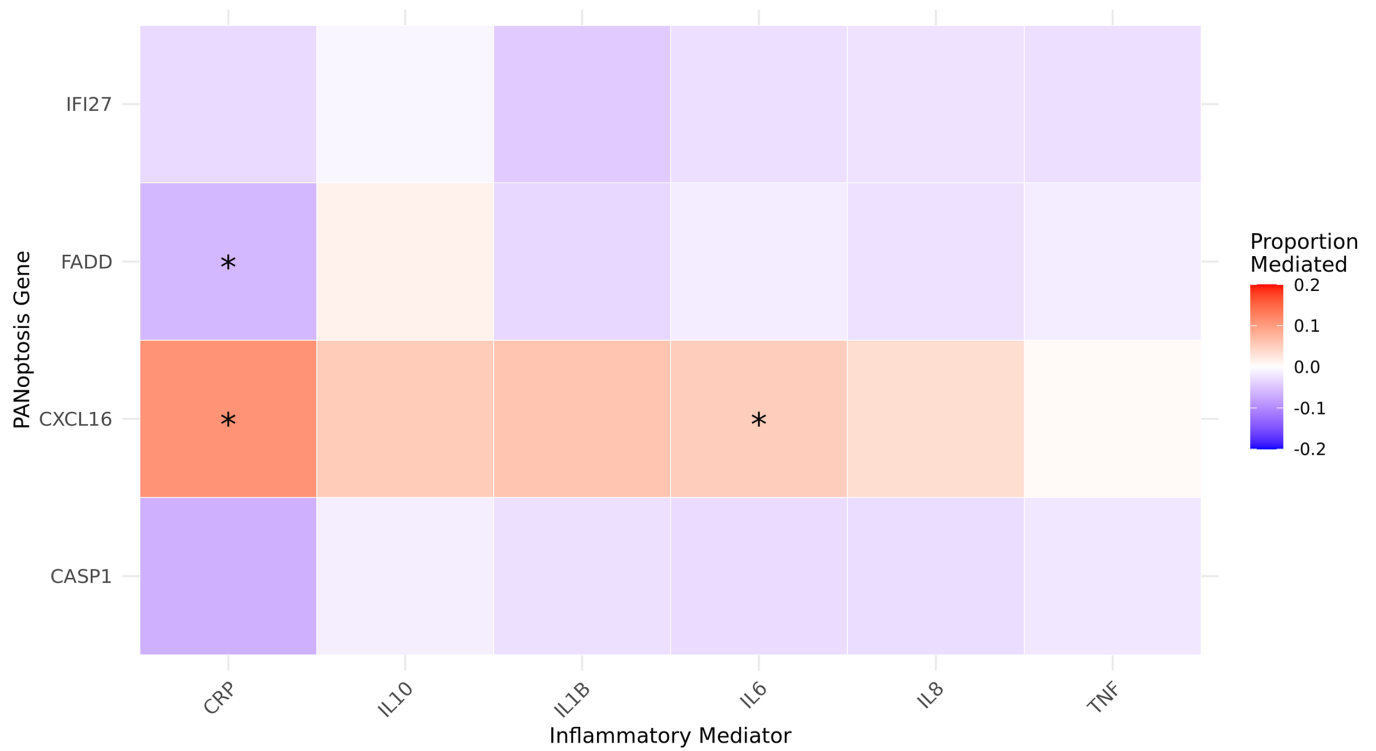

Supplement: Supplementary file 5 — Supplementary Material 5. [file 13041_2026_1324_MOESM5_ESM.pdf]
