# Supplementary material for: Genetically predicted CXCL16 expression is associated with Parkinson’s disease risk and peripheral immune cell dysregulation: a two-sample mendelian randomization study
Source: Mol Brain. 2026 Jun 30;19:52. doi: 10.1186/s13041-026-01324-z (PMC13321530; doi:10.1186/s13041-026-01324-z)
Supplement: Supplementary file 2 — Supplementary Material 2. [file 13041_2026_1324_MOESM2_ESM.pdf]

Supplementary Figure S3

MR Sensitivity Analysis: CASP1 → Parkinson's Disease

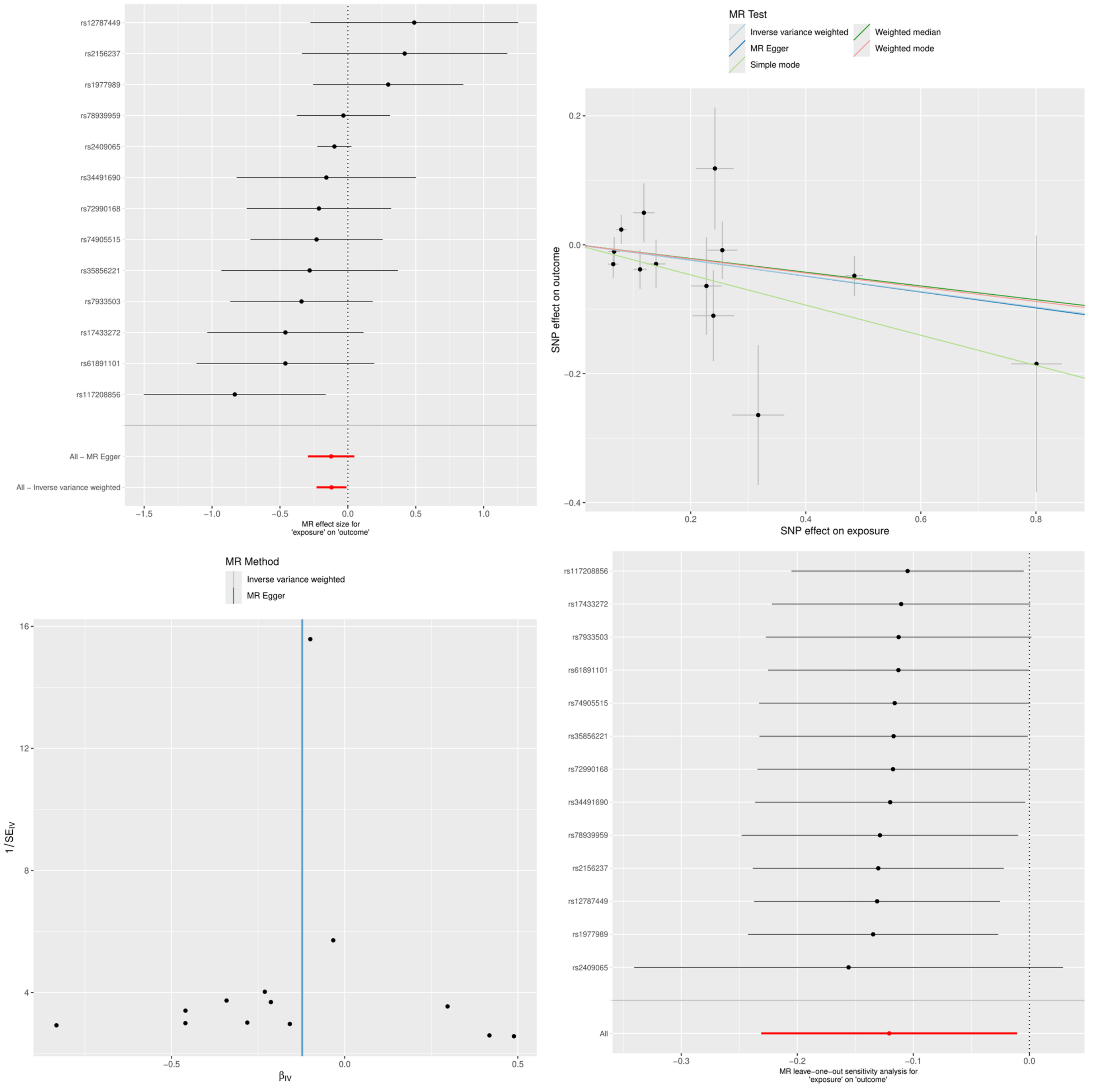

Supplementary Figure S3. MR sensitivity analysis for CASP1 (nSNP=13, mean F=158.7).
Top-left: Forest plot. Top-right: Scatter plot.
Bottom-left: Funnel plot. Bottom-right: Leave-one-out analysis.
IVW: OR=0.886 [0.794–0.989], p=0.032 (nominally significant, exploratory).
MR-Egger intercept p=0.891 (no pleiotropy). MR-PRESSO Global p=0.365 (no outliers).
