# Supplementary material for: Genetically predicted CXCL16 expression is associated with Parkinson’s disease risk and peripheral immune cell dysregulation: a two-sample mendelian randomization study
Source: Mol Brain. 2026 Jun 30;19:52. doi: 10.1186/s13041-026-01324-z (PMC13321530; doi:10.1186/s13041-026-01324-z)
Supplement: Supplementary file 3 — Supplementary Material 3. [file 13041_2026_1324_MOESM3_ESM.pdf]

Supplementary Figure S4

MR Sensitivity Analysis: IFI27 → Parkinson's Disease

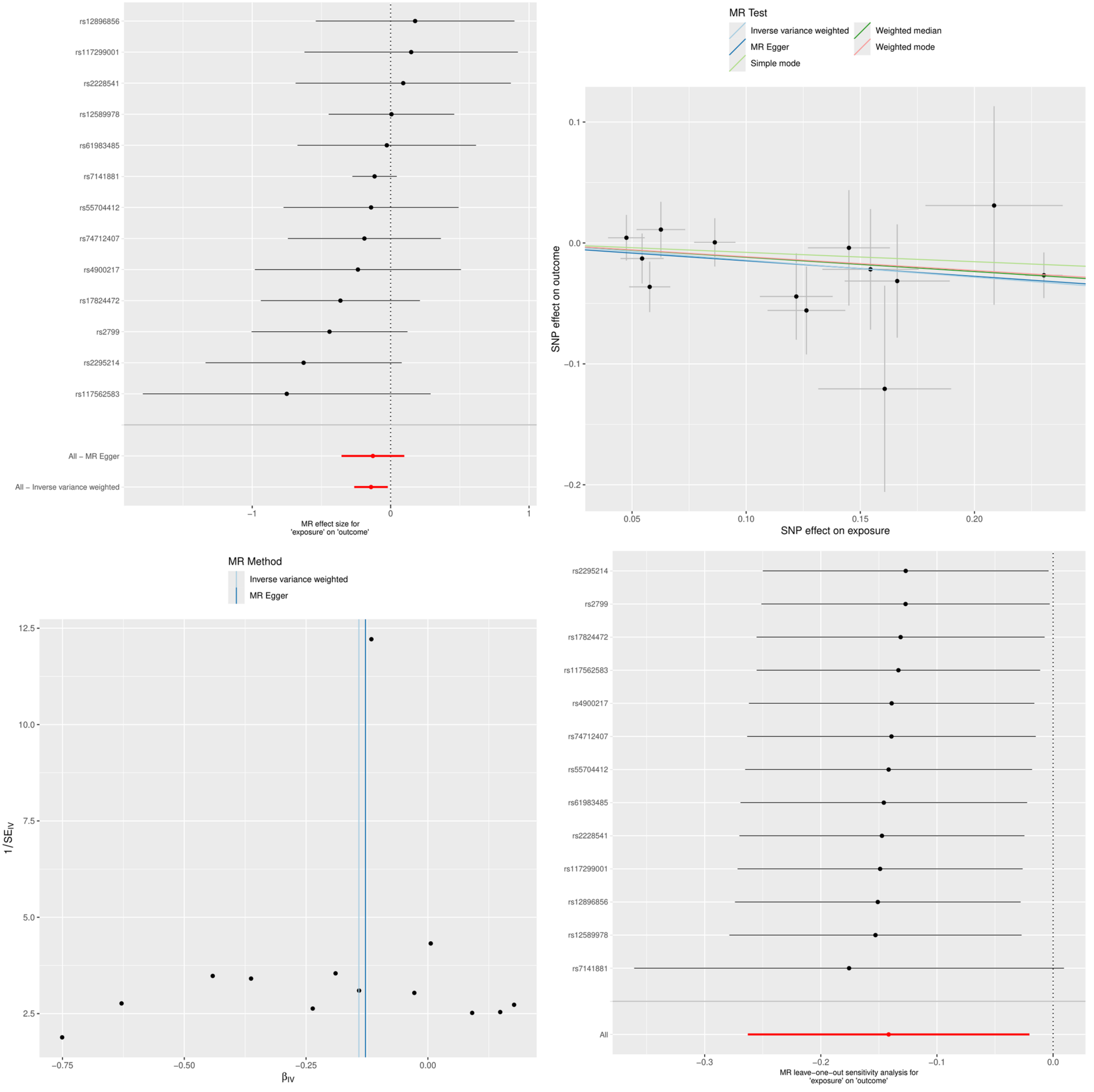

Supplementary Figure S4. MR sensitivity analysis for IFI27 (nSNP=13, mean F=107.1).
Top-left: Forest plot. Top-right: Scatter plot.
Bottom-left: Funnel plot. Bottom-right: Leave-one-out analysis.
IVW: OR=0.868 [0.769–0.980], p=0.022 (nominally significant, exploratory).
MR-Egger intercept p=0.706 (no pleiotropy). MR-PRESSO Global p=0.875 (no outliers).
