# Supplementary material for: Genetically predicted CXCL16 expression is associated with Parkinson’s disease risk and peripheral immune cell dysregulation: a two-sample mendelian randomization study
Source: Mol Brain. 2026 Jun 30;19:52. doi: 10.1186/s13041-026-01324-z (PMC13321530; doi:10.1186/s13041-026-01324-z)
Supplement: Supplementary file 4 — Supplementary Material 4. [file 13041_2026_1324_MOESM4_ESM.pdf]

Supplementary Figure S5

Mediation Analysis: CXCL16 → Immune Traits → Parkinson's Disease

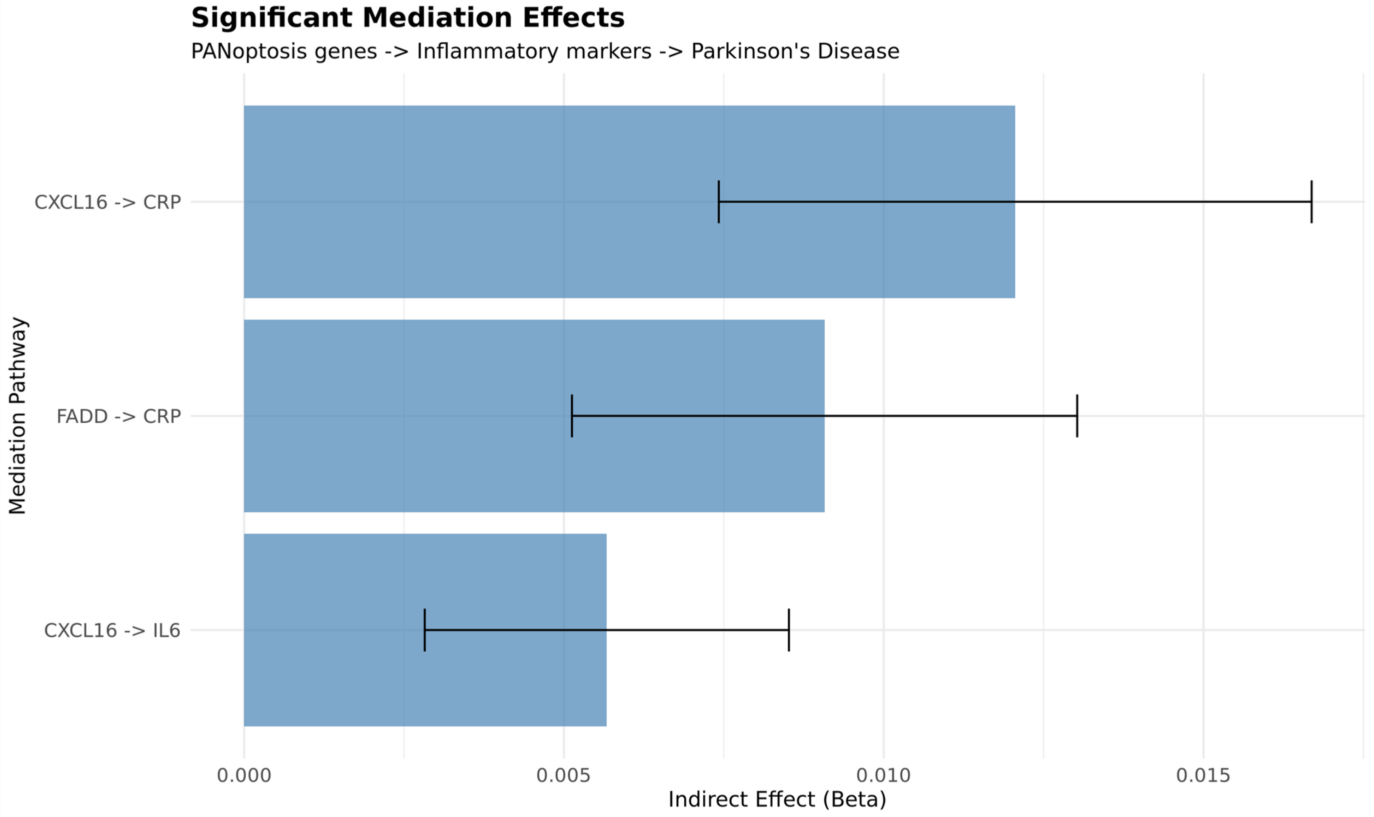

Supplementary Figure S5. Two-step MR mediation analysis results.
Indirect effects ($\beta_1 \times \beta_2$) of CXCL16 expression on PD risk mediated through immune cell phenotypes. Error bars represent 95% CI (delta method).
$SE\_indirect = \sqrt{(\beta_1^2 \times SE_2^2 + \beta_2^2 \times SE_1^2)}$.
Top 20 mediators by |indirect effect| shown. FDR q-values indicated.
63/66 mediators significant at FDR $q < 0.05$.
Full results: Supplementary Table S7.
